# DAGIS Salo cohort profile: a longitudinal and cross-sectional study to identify environmental and individual factors linked to health behaviours, recovery from stress, weight and learning outcomes among Finnish schoolchildren

**DOI:** 10.1186/s12889-026-27007-x

**Published:** 2026-03-19

**Authors:** Josefine Kailaheimo-Björkqvist, Henna Vepsäläinen, Jonna Kesäläinen, Reetta Lehto, Henna Launistola, Jenna Rahkola, Satu Kinnunen, Emmi Tilli, Nanna Wackström, Nithya Serasinghe, Idil Muhumed, Ossian Klingstedt, Eva Roos, Nina Sajaniemi, Maijaliisa Erkkola, Carola Ray

**Affiliations:** 1 Folkhälsan Research Center, Topeliuksenkatu 20, Helsinki, 00250 Finland; 2 Institute of Applied Health Sciences, University of Aberdeen, Foresterhill, Aberdeen, AB25 2ZD UK; 3 Department of Food and Nutrition, University of Helsinki, P.O. Box 66, Helsinki, 00014 Finland; 4 School of Applied Educational Science and Teacher Education, University of Eastern Finland, P.O. Box 111, Joensuu, 80101 Finland; 5 Faculty of Medicine, University of Helsinki, PO BOX 63, Helsinki, 00014 Finland; 6 Faculty of Arts, Psychology and Theology, Akademi University, Tehtaankatu 2, Turku, 20500 Finland; 7 Department of Public Health, University of Helsinki, PO BOX 20, Helsinki, 00014 Finland; 8 Department of Food Studies, Nutrition and Dietetics, Uppsala University, PO Box 560, Uppsala, 751 22 Sweden

**Keywords:** Children, Adolescent, Physical activity, Sleep, Screen time, Food intake, Mental health, Intervention follow-up, Preschool, Socioeconomic status

## Abstract

**Background:**

The global prevalence of childhood obesity has increased significantly, with World Health Organisation emphasising the importance of early childhood for promoting healthy growth and development. In Finland, 29% of boys and 18% of girls aged 2–16 years are living with overweight or obesity. The DAGIS Intervention in 2017–2018 in Southwest Finland aimed to promote healthy energy-balance related behaviours (EBRBs) and self-regulation skills in early childhood education and family settings, hypothesising that improvements would reduce overweight prevalence, increase well-being, and enhance learning outcomes in pre-adolescence. DAGIS Salo is a follow-up study of the DAGIS Intervention, with parallel recruitment of a new cross-sectional sample. This paper describes the DAGIS Salo cohort profile, covering study design, recruitment, measurements, data processing and participant characteristics.

**Methods:**

The DAGIS Salo study (2023–2024) consisted of a larger cross-sectional sample and, within it, a six-year follow-up sample from the DAGIS Intervention. All primary schoolchildren in grades 3–6 in Salo municipality in Southwest Finland and their caregivers were invited to participate, regardless of participation in the DAGIS Intervention. Schoolchildren provided information on movement behaviours (accelerometry), screen use, food consumption, psychosocial well-being, and recovery from stress (heart rate variability). Trained data collectors measured schoolchildren’s anthropometrics and administered learning tests. Caregivers provided information on physical and social home environment and child temperament. School and food service personnel provided information on the school environment.

**Results:**

The DAGIS Salo cohort consists of 540 schoolchildren (27% of eligible participants), including the DAGIS Intervention follow-up sample of 207 schoolchildren (31% of participants from the DAGIS Intervention). The average age of schoolchildren in the cohort was 11 (± 1.15) years and 54% of participants were girls.

**Conclusion:**

The DAGIS Salo study utilised a longitudinal and cross-sectional design, objective measures and validated instruments, and multiple perspectives to examine physical and social environments that influence schoolchildren’s health and behaviour. Study findings can advance equity in schoolchildren’s health, well-being and learning by identifying modifiable factors within early childhood education, home and school environments, to better support EBRBs, well-being and learning of those who need it most.

**Trial registration:**

ISRCTN57165350 (8 January 2015).

**Supplementary Information:**

The online version contains supplementary material available at 10.1186/s12889-026-27007-x.

## Background

The global prevalence of childhood obesity has been rising at an alarming rate. Between 1975 and 2016, the prevalence of children aged 5–19 years living with overweight or obesity quadrupled from 4 to 18% worldwide [1]. In response to this trend, the World Health Organisation (WHO) has identified the early years as a crucial stage for enhancing children’s nutrition, growth and development [1]. Moreover, recent statistics indicate that 29% of boys compared to 18% of girls aged 2–16 years in Finland are living with overweight or obesity [2]. This highlights the need to explore the underlying factors behind these observed sex differences among children living with overweight or obesity. Children’s health, well-being, weight and learning are partly shaped by energy balance-related behaviours (EBRBs) such as food consumption, physical activity (PA), sedentary behaviours and sleep [3, 4]. Importantly, the EBRBs developed in early childhood have been shown to persist into adulthood [1, 5]. Additionally, inadequate self-regulation skills have been associated with unhealthier EBRBs and obesity in children and poor academic achievement in later life [6, 7]. Given that in OECD countries the average enrolment rate in early childhood education and care (ECEC) is 87%, ECEC centres are an important setting for promoting healthy behaviours and reaching children from different socioeconomic backgrounds [8].

The Increased Health and Well-being in Preschools Intervention study (hereafter referred to as the DAGIS Intervention) was a cluster randomised controlled trial in 32 Finnish ECEC centres during 2017–2018 [9]. The DAGIS Intervention aimed to promote healthy EBRBs and self-regulation skills among 3–6-year-old children, hypothesising that these improvements would reduce overweight prevalence, increase well-being, and enhance learning outcomes in pre-adolescence. The 5-month intervention targeted both ECEC centres and homes, focusing on improving self-regulation, PA, and fruit and vegetable consumption, reducing screen time, and lowering sugary food and beverage consumption. The intervention was conducted in Salo and Riihimäki municipalities in Southwest Finland. All 29 public ECEC centres in Salo municipality participated, with 663 children and their caregivers consenting (47% of all eligible participants). Centres were randomised into intervention and control groups. A comprehensive outline of the DAGIS Intervention’s study design and evaluation of short-term study outcomes are published elsewhere [9–11]. Despite some evidence of effective health promotion interventions in ECEC settings [12], there is a need for more studies on the long-term sustainability of these effects [12, 13]. To address this gap, we invited all primary school pupils in grades 3–6 in the Salo municipality to participate in the DAGIS Salo study, with the aim of reaching participants from the DAGIS Intervention in 2017–2018, six years following their initial participation, and to conduct a larger cross-sectional study on school environment, EBRBs and learning.

The aims of the DAGIS Salo study are: to assess how past and present environmental and individual factors are associated with EBRBs and weight among schoolchildren; to identify past and present environmental and individual determinants of self-regulation skills and recovery from stress among schoolchildren; to identify past and present environmental and individual factors linked to learning outcomes among schoolchildren; and to investigate if child sex and family socioeconomic status (SES) moderate the aforementioned associations. In this paper, we describe the DAGIS Salo cohort profile, covering study design, participant recruitment, various measurement methods, data processing and participant characteristics.

## Methods

### Study design

The DAGIS Salo study utilised a dual design, including a larger cross-sectional sample (DAGIS Salo) and within it a longitudinal sample (DAGIS Intervention follow-up). The study included measures for the following levels: school level (questionnaires for principals, teachers, food service personnel and observations of the school environment); family level (questionnaires for both caregivers, if there were two) and child level (objective measures and questionnaires).

### Ethical approval

Ethical approval was granted from the University of Helsinki Ethical Review Board in the Humanities and Social and Behavioural Sciences for both the DAGIS Intervention in 2017–2018 (statement 22/2017) and the DAGIS Salo study in 2023–2024 (statement 46/2023).

### Participants and recruitment

Children who participated in the DAGIS Intervention 2017–2018 were primary schoolchildren in grades 3–6 aged 8–13 years at the time of recruitment for follow-up in 2023–2024. All pupils in grades 3–6 in Salo municipality and their caregivers were eligible to participate in the DAGIS Salo study, independently of whether they had participated in the DAGIS Intervention. Assuming a similar participation rate to the DAGIS Intervention (47%), we estimated around 1000 pupils and their caregivers would participate.

**Fig. 1:**
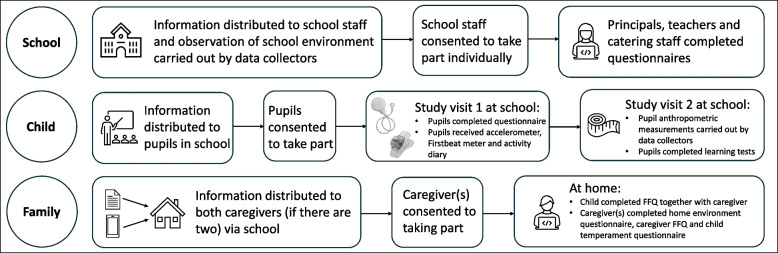
Recruitment and measurements in the DAGIS Salo study by school level, child level and family level

The recruitment and participation procedure are described in Fig. 1. Salo municipality committed to the study, making participation obligatory for all primary schools. The head of the educational department in Salo municipality helped develop the recruitment protocol and data collection took place between October 2023 and April 2024. In accordance with municipality recommendations recruitment information was sent to principals and teachers, who distributed materials to pupils and their caregivers via the electronic communication service between Finnish schools and home (Wilma). Trained data collectors conducted school visits to present the study and deliver paper information materials. Participant information materials, including participant letters, consent forms, infographics, and videos, were made available in Finnish, Swedish and English. Additionally, participant letters and consent forms were translated into Ukrainian for the sizable Ukrainian migrant community in Salo. Informed consent was obtained electronically or via paper consent form from all participating schoolchildren and their caregivers. Both child and caregiver consent were required for child participation and schoolchildren could participate in the study independently of caregivers participating themselves. Measures were uniformly conducted across all schoolchildren who consented to take part. Schoolchildren and caregivers had the choice to participate only in some parts of the study if they so wished, and to withdraw at any point without giving an explanation. Participants who had previously taken part in the DAGIS Intervention were asked for their consent to combine their data from the DAGIS Intervention and DAGIS Salo.

Principals and teachers assisted with organising study visits and delivering information materials and could choose whether to participate in the study themselves. A participant information letter was sent to principals and teachers, inviting them to complete a questionnaire and consent was obtained electronically. Data collectors also visited school food service personnel to deliver paper information letters and consent forms, inviting them to complete the consent form and questionnaire either electronically or on paper form.

All participants were promised feedback to increase motivation to take part and to reward their time. Children received feedback on their health behaviours, including PA, sedentary time, screen use, and food consumption. Caregivers received feedback on their child’s PA and sedentary time, as well as their child’s and their own screen use and food consumption. Schools received group-level feedback. Salo municipality received a summary report of descriptive study results. All participating classes received a diploma for participation and PA equipment. All eligible families entered a lottery, with the chance to win five bowling tickets.

### Measurements

#### School-related factors

Study measures for the DAGIS Intervention and DAGIS Salo study are summarised in Supplementary File 1. An observation of the school environment was conducted by data collectors together with pupils from grades 3–6 according to a standardised protocol. The observation assessed how the school environment and equipment in the school environment promoted physical activity, including an assessment of the indoor physical environment (active learning equipment) and the outdoor physical environments (school playground surface type, playground equipment and recess activity equipment). The observation also assessed the lunch area and school food environment. Principals and teachers completed a questionnaire assessing background factors (age, sex, education, and work experience), their knowledge and attitudes and their training and experience with well-being-related factors in the school environment, including food consumption, PA, sedentary behaviour, sleep and self-regulation. School food service personnel completed a questionnaire assessing background factors, such as age, sex, education level, and work experience in the school kitchen, access to food or nutrition-related training and their opinions on the quality of school lunch and whether they felt they could influence food served in the school.

#### Anthropometry

Children’s weight, height and waist circumference were measured by trained data collectors using a standardised protocol. Children wore light indoor clothing and removed their shoes. Height was measured to the nearest 0.1 cm using a portable stadiometer (Seca 213). Weight was measured to the nearest 0.01 kg using an electronic calibrated scale (Seca 813). Waist circumference was measured to the nearest 0.1 cm using a standard measuring tape. Measurements were repeated twice, and if there was a predetermined difference (> 0.25 kg for weight, > 0.5 cm for height and > 1 cm for waist circumference), a third measurement was taken. International Obesity Task Force (IOTF) [14] and Finnish body mass index z-scores [15], and waist-to-height ratio were calculated from these data.

### Energy balance-related behaviours

#### Movement behaviours

Children’s PA, sedentary time and sleep were measured for seven consecutive days using a triaxial accelerometer (wGT3X-BT, Actigraph, LCC, Pensacola, FL, USA). Participants were instructed how to wear the accelerometer during the first study visit at school. The accelerometers were worn on an adjustable elastic waistband on the right hip. Participants were encouraged to wear the accelerometer continuously, removing it only for showering, sauna, and water-based activities. The waist-worn accelerometer is the most widely used and extensively validated instrument for assessing PA, sedentary time and sleep time in preschool and schoolchildren [16, 17]. Children completed an activity diary for seven days to report wake-up and bedtimes, first and last eating occasion, school times, periods of non-wear, and participation in organised PA.

#### Screen use

Child and caregiver screen use, including time, content and context, were assessed using modified questions from the SCREENS-Q questionnaire [18]. Caregiver and child smartphone screen time and most used applications were assessed via caregiver-reported smartphone logs, using modified questions from the DigiConsumer research project [19] and the SUNRISE Finland Study [20].

#### Food consumption

A 66-item food frequency questionnaire (FFQ) covering nine food groups (vegetables, fruit and berries; dairy products; plant-based drinks, snacks and cheese; fast food at a restaurant and takeaway foods; main courses and cured meats; cereal products; drinks; sweet and savoury treats; and dietary fats) was used to assess child and caregiver food consumption. The previous version of the FFQ, targeted to preschoolers, has shown acceptable relative validity in ranking food group consumption [21] and acceptable reproducibility [22]. It was slightly modified to better represent the commonly used foods among schoolchildren and the current food variety, particularly regarding new plant-based products. The schoolchild was recommended to complete the child FFQ with a caregiver but could also do it independently. Caregivers were also invited to complete a similar FFQ regarding their own diet. The FFQ assessed food consumption over the past seven days, and the response options were not at all; once per week; 2–3 times per week; 4–5 times per week; daily or almost daily (6–7 times per week); 2 times per day; and three times per day or more. Additional questions on special diets, meal patterns, and the use of dietary supplements were included. Caregivers reported the frequency of meals shared with their child and whether they avoided eating certain foods or drinks in the presence of their child.

#### Stress and recovery

The balance between the child’s stress and recovery was measured over four consecutive days via heart rate variability (HRV) [23–25] using the Firstbeat Bodyguard 2 (Firstbeat Technologies, Jyväskylä, Finland) [26]. Participants were instructed on how to wear the device during the first study visit at school.

#### Learning outcomes

Children’s learning capacity was assessed using the Finnish Lukilasse 2 [27] reading comprehension and mathematics tests, standardised and adapted for grades 1–6. Trained data collectors administered the tests during the second study visit in school.

#### Temperament

Children's temperament was assessed using the parent-report format of The Early Adolescent Temperament Questionnaire (EATQ-R), which is a revision of an instrument developed by Capaldi and Rothbart in 1992 [28]. The questionnaire has been designed to map natural disposition and typical patterns of child behaviour.

### Child perspective on home and school environment

The child questionnaire assessed the social and physical home and school environment using instruments appropriate for the age group. The social environment includes role modelling, for example how children perceive their caregivers’ PA, and how often caregivers consume certain foods in the presence of the child; rules at home such as restrictions on screen use and norms relating to eating behaviours, screen use and PA. The physical environment includes availability of foods and screen devices. The child questionnaire included additional items concerning psychosocial well-being, including school satisfaction, loneliness and well-being, which have also been used in the international Health Behaviour in School-aged Children study [29]. Furthermore, three additional questions were included for participating children from grades 5–6; namely, the WHO-5 Well-being Index [30] and the adapted Multidimensional Scale of Perceived Social Support, assessing peer and family support [29, 31]. Frequency of self-reported PA was assessed using the Prochaska physical activity screening measure [32], which is also used in the Finnish LIITU study [33], allowing for national comparison of results. Frequency of outdoor visits was assessed using a modified question from the DAGIS Intervention study [9, 34]. Duration of outdoor and nature visits and activities in nature were assessed using modified questions from studies in children of similar age groups [35–37]. Child nature connectedness was assessed using the Connection to Nature Index [38]. Technoference, i.e. parental use of technological devices that interrupts everyday family relations and interactions, was assessed via the Technoference in Parent–Child Relationships instrument by Stockdale and colleagues [39, 40]. Self-reported frequency of exposure to unhealthy food and beverage advertisements was assessed via a modified version of the question developed by Demers-Potvin and colleagues [41].

### Caregiver perspective on home environment

One of the child’s caregivers was asked to complete a questionnaire regarding background factors, social and physical home environment. Background factors included questions about the child (age, sex) and family SES (parental educational level, employment and occupational status, household income) and demographics such as family composition. Parental educational level (PEL) was then categorised in the following way: ‘low’ = comprehensive school, vocational school, ‘middle’ = polytechnic degree or bachelor’s degree, ‘high’ = master’s degree, licentiate, or doctor. Total household net income was divided by the number of family members using a standard equivalence scales that gives a weight to all members of the household [42].

The social and physical home environment was assessed with items previously used in the DAGIS Survey [43] and the DAGIS Intervention study [10] as well as additional instruments appropriate for the child’s age group (see Supplementary File 1). The social environment includes role modelling, for example how often the caregivers use screen devices or consume certain foods in the presence of the child; rules such as restrictions on screen use and norms relating to eating behaviours and PA. Food parenting practices were assessed using items from the validated HomeSTEAD family food practices survey [44]. The PA climate and family norms related to PA were assessed using the Family Health Climate scale (FHC–PA) developed by Niermann and colleagues [45]. The physical environment includes availability of screen devices and availability of foods (e.g. fruit and vegetables and sugary foods and drinks), accessibility of PA promoting and natural environments and perceived barriers for PA, and vegetable and fruit consumption. Frequency, duration and activities during parent–child nature visits were assessed using the same instruments as mentioned above for the child questionnaire. The validated Strength and Difficulties Questionnaire (SDQ) was used to assess child emotional and behavioural difficulties including emotional symptoms, conduct problems, hyperactivity-inattention, peer problems and prosocial behaviour [46]. Parental happiness was assessed using the self-reported Subjective Happiness Scale [47]. The second caregiver, if there was one, was asked to complete a shorter version of the questionnaire, which included most of the questions concerning social and physical home environment to investigate the home environment from the perspective of the second caregiver.

### Data processing, analysis and dissemination

Data processing, analysis and manuscript writing will follow during 2026. All participants in the study were pseudonymised using an identification number. Data from paper questionnaires and activity diaries was entered manually according to a predefined data input protocol. Data was cleaned in R version 4.4.1 (R Core Team, 2022). This included the removal of duplicates and standardisation of variable formats. For essential missing or ambiguous data, such as child’s date of birth or PEL, the caregiver was contacted via e-mail or phone.

Child participant records from the DAGIS Intervention and DAGIS Salo were combined. The linkage was first performed automatically using the child’s full name and date of birth. All non-connected records were checked manually, as mismatches could occur from multiple reported names, changes in last name or errors when entering date of birth. Potential matches were verified using additional information, such as caregiver’s name and family address. Inconsistencies in the date of birth were clarified by e-mail with caregivers.

Raw accelerometer data (ActiGraph.csv files) was processed using R-package GGIR [48] to create variables describing PA intensities and sleep-related factors. The accelerometer and HRV data from Firstbeat were preprocessed using R to create a format suitable for activity and HRV analysis with the MATLAB Biosignal Toolkit. The accelerometer data was processed into 1-min segments, while the HRV data was processed into both 1-min and 5-min 35-s segments. Afterwards, the data was processed in R to enable integration with other data sources for comprehensive analysis.

We will use multilevel regression models, and linear mixed models in longitudinal analyses. In addition, we will investigate moderation effects by child sex and family SES. We will use SPSS and R statistical packages when conducting the analyses. Results will be disseminated to researchers and stakeholders via scientific publications, oral presentations, press releases and social media communication. Communication related to the project will be made available on the project website: http://dagis.fi.

## Results

**Fig. 2:**
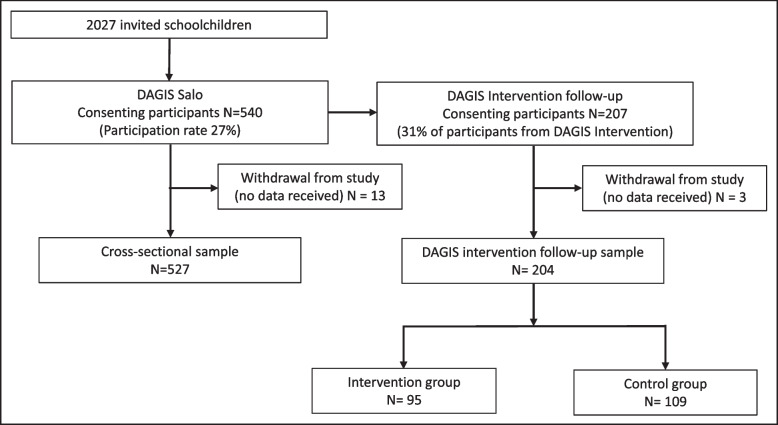
Flow chart of participating schoolchildren in the DAGIS Salo study. DAGIS Intervention follow-up sample included in cross-sectional sample

The flow chart of participating schoolchildren in the DAGIS Salo study is presented in Fig. 2. The DAGIS Salo cohort consists of 540 children (27% of eligible participants; 33% of grade 3 pupils, 25% of grade 4 pupils, 28% of grade 5 pupils and 21% of grade 6 pupils). Included within the cohort is the DAGIS Intervention follow-up sample of 207 children; 31% of participants from DAGIS Intervention in 2017–2018. A total of 460 first caregivers and 56 s caregivers gave consent to participate themselves. Caregivers who completed the caregiver questionnaire were predominately mothers (82%). All 22 primary schools in Salo municipality participated in the study, including smaller rural schools and larger schools in urban areas.

Table 1 describes characteristics of study participants. The average age of children in the DAGIS Salo cohort was 11 (± 1.15) years and 54% of participants were girls. BMI categories with IOTF references [14] indicate that 11% were underweight, 71% normal weight, 15% overweight and 3% obese. BMI categories with Finnish references [44] are included in Supplementary File 2 to allow national comparison.

**Table 1:** Characteristics of study participants

Study participants (children)	Cross-sectional sample		Follow-up sample (included in cross-sectional sample)	
	*N* = 540		*N* = 207	
Child age (years)				
Mean ± SD	11.0 ± 1.15		11.4 ± 1.00	
Minimum—maximum	8.85—14.02		9.11—13.15	
Missing	6^c^			
Child sex	**n**	**%**	**n**	**%**
Female	289	54	108	52
Male	250	46	98	48
Missing	1^c^			
BMI categories (IOTF)				
Underweight	51	11	18	10
Normal weight	330	71	129	72
Overweight	67	15	29	16
Obese	15	3	4	2
Missing	77^c^			
Highest education level in the family^a^				
Low	142	28	52	26
Middle	227	45	96	48
High	137	27	53	26
Missing	34^c^			
Household relative income tertiles^b^				
Low	115	33	40	28
Middle	115	33	48	34
High	114	33	55	38
Missing	26^d^			
Family type (the child lives with…)				
Both parents in one home	284	78	118	79
Weekly turns with both parents	23	6	12	8
Primarily with the mother	54	15	18	12
Primarily with the father	2	1	1	1
In foster care/other family member	2	1	1	1
Missing	5^d^			
Child grade				
3	159	29	28	14
4	125	23	44	21
5	144	27	71	34
6	112	21	64	31
School size of child participants				
Small (< 100 pupils)	133	25	46	22
Medium (100–299 pupils)	269	50	113	55
Large (≥ 300 pupils)	138	25	48	23

^a^Categories of parental educational level; ‘low’ = comprehensive school, vocational school, ‘middle’ = polytechnic degree or bachelor’s degree, ‘high’ = master’s degree or licentiate/doctoral degree

^b^Total household net income divided by the number of family members using a standard equivalence scale. Cut-offs for relative income tertiles; less than EUR 1904.7 = ‘low’, EUR 1904.7 < > EUR 2976 = ‘middle’, higher than EUR 2976 = ‘high’

^c^Base for calculations: 540 children with consent

^d^Base for calculation: 370 caregivers with consent who answered the caregiver questionnaire

Table 2 shows data collected from consented participants by child, family, and school level. Data availability was highest for school and child level data and lower for family level data, specifically data from the second caregiver.

**Table 2:** Data collected from consented participants by child, family, and school level

Study measure	Cross-sectional sample	Follow-up sample (included in cross-sectional sample)
	**N (%)**	**N (%)**
Child level^a^		
Child questionnaire	500 (93%)	192 (93%)
Activity diary	483 (89%)	186 (90%)
First Beat data	409 (76%)	158 (76%)
Actigraph data^b^	413 (76%)	163 (79%)
Child FFQ	420 (78%)	164 (79%)
Child temperament	375 (69%)	154 (74%)
Learning tests	448 (83%)	180 (87%)
Anthropometric data	469 (87%)	180 (87%)
Family level^c,d^		
Caregiver 1 questionnaire	370 (80%)	153 (84%)
Caregiver 2 questionnaire	47 (84%)	23 (85%)
Caregiver 1 FFQ	340 (74%)	135 (74%)
Caregiver 2 FFQ	41 (73%)	21 (78%)
School level^e,f^		
Observation of school environment	22 (100%)	
Principal questionnaire	18 (82%)	
Teacher questionnaire	51 (42%)	
Food service personnel questionnaire	21 (96%)	

^a^Base for calculations = 540 children with consent to take part; 207 children in follow-up sample

^b^Data available for at least four days, of which one was a weekend day

^c^Base for calculations = 440 first caregivers consented to taking part themselves; 182 in follow-up sample

^d^Base for calculations = 56 s caregivers consented to taking part themselves; 27 in follow-up sample

^e^Base for calculations = 22 primary schools in Salo municipality

^f^Base for calculations = 122 primary school teachers in grades 3–6 in Salo municipality

## Discussion

The DAGIS Salo study successfully reached participants from the DAGIS intervention, six years after their initial involvement, while simultaneously recruiting a new cross-sectional sample of schoolchildren. All primary schools (grades 3–6) within the Salo municipality participated, from large urban schools to medium-sized and smaller rural schools. This study has collected data from multiple perspectives on schoolchildren’s health, well-being, and learning, enabling us to address our primary research questions in upcoming analyses. Furthermore, through school-based recruitment we reached a broad representation of participants across various socioeconomic backgrounds and girls and boys were equally represented in the study sample, facilitating future exploration of differing associations according to child sex and family SES.

This study has several strengths. Firstly, its longitudinal design will advance understanding of how early-life factors, including social and physical environments, influence later child EBRBs, well-being, weight, and learning. The distribution of family SES was similar to the DAGIS Intervention [10] and we retained an equal number of participants from the intervention and control group of the DAGIS intervention, enabling the investigation of long-term effects of the DAGIS intervention. Moreover, we examine physical and social factors within home and school environments from multiple perspectives, including schoolchildren, caregivers, principals, teachers, and school food service personnel. Understanding a child's temperament, the stability of their temperament, and factors influencing their behaviour can help teachers and caregivers tailor their approaches to better support children’s needs and enhance their learning and success in school.

Additionally, the study employs objective measures for movement behaviours, including novel measures in children for recovery from stress. The accelerometer data collected in the study can be harmonised with other datasets, enabling cross-regional comparisons of children’s movement behaviours. Furthermore, the study outcomes align with the core outcome set for childhood obesity prevention developed by Brown and colleagues, encompassing anthropometry, food consumption, PA, sedentary behaviour, sleep, child well-being, parenting practices, home and ECEC centre environment [49]. Finally, the use of validated instruments such as the SDQ [46] and items from the international Health Behaviour in School-aged Children study [29] facilitates both domestic and international comparative studies.

A limitation in this study is the relatively low participation rate. Participation was higher for grades 5 and 6 in the follow-up sample (34% and 31% respectively) compared to grades 3 and 4 (14% and 21% respectively). This corresponds to the participation rates in the DAGIS Intervention; 30% for 5-year-olds and 32% for 6-year-olds compared to 17% for 3-year-olds and 21% for 4-year-olds. Furthermore, DAGIS Intervention participants who gave negative consent for follow-up in this study (N = 60) had lower family SES at the time of the DAGIS Intervention compared to those who provided positive consent. The attrition of participants from a lower SES background may bias results; however, the final distribution of family SES in this longitudinal and cross-sectional study was still similar to the DAGIS Intervention [10].

Participation rates in studies investigating schoolchildren’s health behaviours have been steadily decreasing in recent years, both in Finland and globally [50]. This study faced similar recruitment challenges. Possible reasons for this trend may include the high volume of school-based studies, a lack of school personnel, and a perception by school staff that they do not receive enough incentives or timely study results [50]. Moreover, the participant burden for schoolchildren may have been perceived as high due to multiple study measures. To mitigate this, most child measures were scheduled during school hours. Similarly, the perceived burden for participating caregivers may have discouraged them from taking part or led to missing data. To address these issues, feedback for children, caregivers, and school staff was used as an incentive to increase the motivation among school staff to promote the study and to encourage pupils and caregivers to participate. However, delivering feedback to all study participants within the same academic year required substantial resources. Therefore, researchers planning future studies should include costs for staff time in grant proposals and automate the feedback process, where possible, to ensure the timely delivery of feedback to study participants.

We utilised more resources than planned to recruit participants for this study. The municipality initially recommended using only the electronic communication service between Finnish schools and home (Wilma) to recruit participants; however, based on low participation rates in autumn 2023 (below 20% of eligible participants) we adapted new recruitment strategies to increase our participation rate. Trained data collectors made in-class visits to promote the study, familiarise pupils with the accelerometers and Firstbeat meters, and to answer any pupil questions regarding the different study measures. In addition, posters were used to promote the study in schools as well as community spaces such as youth and sport centres. Mainstream media helped promote the study to caregivers via the local newspaper, and targeted videos and infographics were used to promote the study via social media to children, caregivers, and school staff in Salo municipality. Finally, to lower the threshold for caregiver participation, we provided caregivers with paper questionnaires and the option to complete the questionnaires either electronically or on paper, recognising that the perceived burden of completing electronic questionnaires may be higher for some participants due to the high amount of electronic communication.

Despite these efforts, we particularly struggled to obtain responses from the second caregiver, who were predominately fathers in this study. The response rate was similar to the DAGIS Intervention, where only 8% of persons who completed the caregiver questionnaire were fathers [10]. Fathers are frequently under-represented in child health research [51], and men are generally less likely to participate in longitudinal health research [52]. For example, a systematic review on the participation of fathers in observational studies on parenting and childhood obesity and obesity-related health behaviours identified 667 studies, of which only 17% of the total participants were fathers [53]. The sustained under-representation of fathers in child health research is problematic as it hinders investigation into fathers’ influence on children’s health behaviours [51, 53]. Future observational studies and interventions targeting child and adolescent health should investigate the effectiveness of targeted strategies to recruit fathers. We also recommend simplifying the process for caregiver participation by integrating the consent form and caregiver questionnaire into the same form and providing caregivers with forms both electronically and on paper format.

## Conclusion

Despite challenges with recruitment, the DAGIS Salo study successfully engaged participants from the DAGIS Intervention, while simultaneously recruiting a new cohort of schoolchildren. The study collected comprehensive data from a variety of stakeholders, with a particular emphasis on schoolchildren themselves, who provided information on multiple health behaviours, recovery from stress, and learning outcomes. Additionally, data was gathered on a broad range of determinants, including child temperament, physical environmental factors, and social environmental factors such as norms, attitudes, and role modelling. The findings derived from this data have the potential to advance equality in children’s health by recognising modifiable factors within the ECEC, school and home environment, to better support the EBRBs, well-being and learning of those who need it most.

## Supplementary Information

Supplementary Material 1.

Supplementary Material 2.

## Data Availability

Data will be made available upon reasonable request. Researchers interested in the data from this study may contact the DAGIS consortium leader Eva.Roos@folkhalsan.fi.

## Abbreviations

EATQ-R: Early Adolescent Temperament Questionnaire
ECEC: Early childhood education and care
EBRB: Energy balance-related behaviours
FFQ: Food frequency questionnaire
HRV: Heart rate variability
IOTF: International Obesity Task Force
OECD: Organisation for Economic Co-operation and Development
PA: Physical activity
PEL: Parental educational level
SDQ: Strength and Difficulties Questionnaire
SES: Socioeconomic status
