# Supplementary material for: DAGIS Salo cohort profile: a longitudinal and cross-sectional study to identify environmental and individual factors linked to health behaviours, recovery from stress, weight and learning outcomes among Finnish schoolchildren
Source: BMC Public Health. 2026 Mar 19;26:1384. doi: 10.1186/s12889-026-27007-x (PMC13123094; doi:10.1186/s12889-026-27007-x)
Supplement: Supplementary file 1 — Supplementary Material 1. [file 12889_2026_27007_MOESM1_ESM.pdf]

Supplementary File 1: Summary of study measures in the DAGIS Intervention and DAGIS Salo study

Variable	Measure	Dagis Intervention	DAGIS Salo	Data source	References
**Child level**<br>Child characteristics	Age, sex, birth order, family type, ECEC centre	✓	✓	Caregiver-reported	
Child movement behaviours	PA, sedentary time and sleep via 24 hours/7-day waist-worn accelerometer (wGT3X-BT, Actigraph)	✓	✓	Objective measure	(1,2)
	Participation in organised PA, wake-up and bedtimes via 7-day PA and sleep diary		✓	Child-reported	
	PA norms and attitudes via questionnaire	✓	✓	Caregiver and child-reported	(3,4)
Child screen use	Screen time via screen diary	✓		Caregiver-reported	(5,6)
	Screen time, content and context via SCREENS-Q		✓	Child-reported	(7)
	Screen use norms via questionnaire	✓	✓	Caregiver and child-reported	(7,8)
	Smartphone time and app use via smartphone log		✓	Objective measure, caregiver-reported	(9,10)
Child outdoor visits and nature connection	Frequency of outdoor visits	✓	✓	Caregiver and child-reported	(11,12)
	Activities in nature and duration of nature visits via questionnaire		✓	Child-reported	(13–15)
	Connection to nature via Connection to Nature Index		✓	Child-reported	(16)
Child food consumption, attitudes and norms	Food consumption via FFQ	✓	✓	Child and caregiver-reported	(6,17)
	Food norms and attitudes via questionnaire	✓	✓	Child and caregiver-reported	(4)
	Digital food environment and exposure to advertising via questionnaire		✓	Child-reported	(18)
Child temperament	Temperament via behaviour questionnaire	✓	✓	Caregiver-reported	(19)
Child anthropometrics	Height, weight and waist circumference	✓	✓	Caregiver-reported, Researcher-measured	
Child recovery from stress	HRV via Firstbeat Bodyguard 2		✓	Objective measure	(20–23)
	Emotional and behavioural difficulties via SDQ		✓	Caregiver-reported	(24)

Child psychosocial well-being	Self-reported health and well-being, loneliness, social support and school satisfaction via questionnaire		✓	Child-reported	(4,25,26)
Child learning skills	Reading and arithmetic skills via Lukilasse 2		✓	Researcher-administered	(27)
**Family level**<br>Caregiver characteristics	Age, sex, educational status, household income, work status, work time	✓	✓	Caregiver-reported	
Family environment	Parental role modelling, knowledge, norms and attitudes for health behaviours, availability of food items and screens, accessibility of food items, screens and PA-promoting environments via questionnaires	✓	✓	Caregiver-reported and child-reported	(8,12,28–33)
Caregiver screen use	Screen time and content via SCREEENS-Q		✓	Caregiver-reported	(7)
	Smartphone time and app use via smartphone log		✓	Objective measure, caregiver-reported	(9,10)
	Technoference via Technoference in Parent-Child Relationship instrument		✓	Child-reported	(34,35)
Caregiver outdoor visits	Frequency of family outdoor visits together with the child via questionnaire	✓	✓	Caregiver-reported	(13–15)
	Activities in nature and duration of nature visits via questionnaire		✓	Caregiver-reported	(13–15)
Caregiver food consumption	Food consumption via FFQ	✓	✓	Caregiver-reported	(6,17)
Caregiver happiness	Happiness via Subjective Happiness Scale		✓	Caregiver-reported	(36)
**ECEC and school level**<br>Educational facility characteristics	Size, number of personnel, rural/urban	✓	✓	Educator-reported	
Educator characteristics	Age, sex, education and work experience	✓	✓	Educator-reported	
ECEC centre and school environment	Observation of PA-promoting indoor and outdoor educational spaces and food canteen	✓	✓	Researcher-reported	
	Educator knowledge, attitudes, training and experience with well-being-related factors via questionnaire	✓	✓	Educator-reported	(31,37)
	Social school environment via questionnaire		✓	Child-reported	(38)
	Attitudes and practices concerning food and eating at ECEC centre/school and food related training	✓	✓	Food-service personnel and educator-reported	(37,39,40)

References

1. Lynch BA, Kaufman TK, Rajjo TI, Mohammed K, Kumar S, Murad MH, et al. Accuracy of Accelerometers for Measuring Physical Activity and Levels of Sedentary Behavior in Children: A Systematic Review. J Prim Care Community Health. 2019 Sep 11;10:2150132719874252.

2. Smith C, Galland B, Taylor R, Meredith-Jones K. ActiGraph GT3X+ and Actical Wrist and Hip Worn Accelerometers for Sleep and Wake Indices in Young Children Using an Automated Algorithm: Validation With Polysomnography. Front Psychiatry. 2020 Jan 14;10:958.

3. Prochaska JJ, Sallis JF, Long B. A Physical Activity Screening Measure for Use With Adolescents in Primary Care. Archives of Pediatrics & Adolescent Medicine. 2001 May 1;155(5):554–9.

4. Inchley J, Currie D, Samdal O, Jåstad A, Cosma A, Nic Gabhainn S. Health Behaviour in School-aged Children (HBSC) Study Protocol: background, methodology and mandatory items for the 2021/22 survey. Glasgow: MRC/CSO Social and Public Health Sciences Unit, University of Glasgow.; 2023.

5. Wen LM, Ploeg HP van der, Kite J, Cashmore A, Rissel C. A Validation Study of Assessing Physical Activity and Sedentary Behavior in Children Aged 3 to 5 Years. 2010 Aug 1 [cited 2025 Jun 25]; Available from: https://journals.humankinetics.com/view/journals/pes/22/3/article-p408.xml

6. Määttä S, Vepsäläinen H, Lehto R, Erkkola M, Roos E, Ray C. Reproducibility of Preschool Personnel and Guardian Reports on Energy Balance-Related Behaviors and Their Correlates in Finnish Preschool Children. Children. 2018 Nov;5(11):144.

7. Klakk H, Wester CT, Olesen LG, Rasmussen MG, Kristensen PL, Pedersen J, et al. The development of a questionnaire to assess leisure time screen-based media use and its proximal correlates in children (SCREENS-Q). BMC Public Health. 2020 May 12;20(1):664.

8. Ray C, Figuereido R, Vepsäläinen H, Lehto R, Pajulahti R, Skaffari E, et al. Effects of the Preschool-Based Family-Involving DAGIS Intervention Program on Children's Energy Balance-Related Behaviors and Self-Regulation Skills: A Clustered Randomized Controlled Trial. Nutrients. 2020 Aug 26;12(9).

9. DigiConsumers Researchers. DigiConsumers [Internet]. 2023 [cited 2023 Oct 10]. Available from: https://digiconsumers.fi/en/what-is-digiconsumers/

10. Engberg E, Ojala A, Paasio H, Lahti J, Koski P, Vehviläinen-Julkunen K, et al. Sociodemographic factors, parental mental health and movement behaviours in the early years: the SUNRISE Finland study protocol. Journal of Activity, Sedentary and Sleep Behaviors. 2024 Jan 3;3(1):3.

11. Määttä S, Lehto R, Nislin M, Ray C, Erkkola M, Sajaniemi N, et al. Increased health and well-being in preschools (DAGIS): rationale and design for a randomized controlled trial. BMC Public Health. 2015 Apr 18;15(1):402.

12. Ray C, Kaukonen R, Lehto E, Vepsäläinen H, Sajaniemi N, Erkkola M, et al. Development of the DAGIS intervention study: a preschool-based family-involving study promoting preschoolers' energy balance-related behaviours and self-regulation skills. BMC Public Health. 2019 Dec 12;19(1):1670.

13. Schmidt T. Danske børns aktiviteter og ophold i naturen. Basisdata fra Center for Børn og Naturs baselineundersøgelse 2018/19 [Internet]. 2020 [cited 2023 Oct 10]. Available from: https://centerforboernognatur.dk/grafik-og-billeder/CfBN_Rapport_Danske_b_rns_aktiviteter_og_ophold_i_naturen_WEB_final.pdf

14. Neuvonen M, Lankia T, Kangas K, Koivula J, Nieminen M, Sepponen AM, et al. Luonnon virkistyskäyttö 2020. Luonnonvara- ja biotalouden tutkimus 41/2022 [Internet]. Helsinki: Luonnonvarakeskus; 2022. Available from: http://urn.fi/URN:ISBN:978-952-380-429-6

15. Garrett J, Wheeler B, Akbari A, Fry R, Geary R, Lovell R, et al. Association between greenspace and time spent in nature with subjective wellbeing: a cross-sectional data linkage study. The Lancet. 2021 Nov 1;398:S47.

16. Cheng JCH, Monroe MC. Connection to Nature: Children's Affective Attitude Toward Nature. Environment and Behavior. 2012 Jan 1;44(1):31–49.

17. Korkalo L, Vepsäläinen H, Ray C, Skaffari E, Lehto R, Hauta-Alus HH, et al. Parents' Reports of Preschoolers' Diets: Relative Validity of a Food Frequency Questionnaire and Dietary Patterns. Nutrients. 2019 Jan 13;11(1).

18. Demers-Potvin É, White M, Potvin Kent M, Nieto C, White CM, Zheng X, et al. Adolescents' media usage and self-reported exposure to advertising across six countries: implications for less healthy food and beverage marketing. BMJ Open. 2022 May 1;12(5):e058913.

19. Capaldi DM, Rothbart MK. Development and Validation of an Early Adolescent Temperament Measure. Journal of Early Adolescence. 1992;12:153-173. Translated to Finnish by Katri Raikkonen-Talvitie and the Developmental Psychology Research Group of University of Helsinki.

20. Speer KE, Naumovski N, McKune AJ. Heart rate variability to track autonomic nervous system health in young children: Effects of physical activity and cardiometabolic risk factors. Physiology & Behavior. 2024 Jul 1;281:114576.

21. Speer KE, Semple S, Naumovski N, McKune AJ. Measuring Heart Rate Variability Using Commercially Available Devices in Healthy Children: A Validity and Reliability Study. Eur J Investig Health Psychol Educ. 2020 Jan 10;10(1):390–404.

22. Kim HJ, Park Y, Lee J. The Validity of Heart Rate Variability (HRV) in Educational Research and a Synthesis of Recommendations. Educ Psychol Rev. 2024 Apr 11;36(2):42.

23. Parak J, Korhonen I. Accuracy of Firstbeat Bodyguard 2 beat-to beat heart rate monitor [Internet]. Tampere: Tampere University of Technology; 2014 [cited 2025 Jun 25]. Available from: https://www.firstbeat.com/wp-content/uploads/2015/10/white_paper_bodyguard2_final.pdf

24. Goodman R. The Strengths and Difficulties Questionnaire: A Research Note. Journal of Child Psychology and Psychiatry. 1997;38:581–6.

25. Topp CW, Østergaard SD, Søndergaard S, Bech P. The WHO-5 Well-Being Index: A Systematic Review of the Literature. Psychotherapy and Psychosomatics. 2015 Mar 28;84(3):167–76.

26. Zimet GD, Dahlem NW, Zimet SG, Farley GK. The Multidimensional Scale of Perceived Social Support. Journal of Personality Assessment. 1988 Mar 1;52(1):30–41.

27. Häyrinen T, Serenius-Sirve S, Korkman M. Lukemis-, kirjoittamis- ja laskemisvalmiuksien arviointi. Helsinki: Hogrefe Psykologien Kustannus Oy; 2013.

28. Niermann C, Krapf F, Renner B, Reiner M, Woll A. Family health climate scale (FHC-scale): development and validation. International Journal of Behavioral Nutrition and Physical Activity. 2014 Mar 5;11(1):30.

29. Pinard CA, Yaroch AL, Hart MH, Serrano EL, McFerren MM, Estabrooks PA. The Validity and Reliability of the Comprehensive Home Environment Survey (CHES). Health Promotion Practice. 2014 Jan 1;15(1):109–17.

30. Mouratidou T, Miguel ML, Androutsos O, Manios Y, De Bourdeaudhuij I, Cardon G, et al. Tools, harmonization and standardization procedures of the impact and outcome evaluation indices obtained during a kindergarten-based, family-involved intervention to prevent obesity in early childhood: the ToyBox-study. Obesity Reviews. 2014;15(S3):53–60.

31. Manios Y, Androutsos O, Katsarou C, Iotova V, Socha P, Geyer C, et al. Designing and implementing a kindergarten-based, family-involved intervention to prevent obesity in early childhood: the ToyBox-study. Obesity Reviews. 2014;15(S3):5–13.

32. González-Gil EM, Mouratidou T, Cardon G, Androutsos O, De Bourdeaudhuij I, Góźdź M, et al. Reliability of primary caregivers reports on lifestyle behaviours of European pre-school children: the ToyBox-study. Obesity Reviews. 2014;15(S3):61–6.

33. Vaughn AE, Dearth-Wesley T, Tabak RG, Bryant M, Ward DS. Development of a Comprehensive Assessment of Food Parenting Practices: The Home Self-Administered Tool for Environmental Assessment of Activity and Diet Family Food Practices Survey. J Acad Nutr Diet. 2017 Feb;117(2):214–27.

34. McDaniel BT, Coyne SM. “Technoference”: The interference of technology in couple relationships and implications for women’s personal and relational well-being. Psychology of Popular Media Culture. 2016;5:85–98.

35. Stockdale LA, Coyne SM, Padilla-Walker LM. Parent and Child Technoference and socioemotional behavioral outcomes: A nationally representative study of 10- to 20-year-Old adolescents. Computers in Human Behavior. 2018 Nov 1;88:219–26.

36. Lyubomirsky S, Lepper HS. A Measure of Subjective Happiness: Preliminary Reliability and Construct Validation. Social Indicators Research. 1999 Feb 1;46(2):137–55.

37. Benjamin SE, Ammerman AS, Ward DS, Ball SC, Sommers JK, Molloy M, et al. An Intervention to Promote Healthy Weight: Nutrition and Physical Activity Self-Assessment for Child Care (NAP SACC) Theory and Design. [cited 2025 Jun 25]; Available from: https://stacks.cdc.gov/view/cdc/20150

38. Kokko S, Martin L. Lasten ja nuorten liikuntakäyttäytyminen Suomessa, LIITU-tutkimuksen tuloksia 2022. Valtion liikuntaneuvoston julkaisuja; 2023.

39. Ruokakasvatusyhdistys Ruukun. Ruokailun ja ruokakasvatuksen nykytila [the current state of food education] [Internet]. 2023 [cited 2023 Oct 10]. Available from: https://nykytila.fi/

40. Laitinen AL, Antikainen A, Mikkonen S, Kähkönen K, Talvia S, Varjonen S, et al. The 'Tasty School' model is feasible for food education in primary schools. Journal of Human Nutrition and Dietetics. 2023;36(1):75–85.
