# Supplementary material for: DAGIS Salo cohort profile: a longitudinal and cross-sectional study to identify environmental and individual factors linked to health behaviours, recovery from stress, weight and learning outcomes among Finnish schoolchildren
Source: BMC Public Health. 2026 Mar 19;26:1384. doi: 10.1186/s12889-026-27007-x (PMC13123094; doi:10.1186/s12889-026-27007-x)
Supplement: Supplementary file 2 — Supplementary Material 2. [file 12889_2026_27007_MOESM2_ESM.pdf]

Supplementary File 2: BMI categories with Finnish references.

BMI categories with Finnish references	Cross-sectional sample		Follow-up sample (included in cross-sectional sample)	
	N	%	N	%
Severe underweight	5	1	0	0
Underweight	14	3	8	4
Normal weight	364	79	141	78
Overweight	65	14	25	14
Obese	15	3	6	3
Missing	77[a]			

a Base for calculations: 540 children with consent.
